# Day-to-Day Variability and Year-to-Year Reproducibility of Accelerometer-Measured Free-Living Sit-to-Stand Transitions Volume and Intensity among Community-Dwelling Older Adults

**DOI:** 10.3390/s21186068

**Published:** 2021-09-10

**Authors:** Antti Löppönen, Laura Karavirta, Erja Portegijs, Kaisa Koivunen, Taina Rantanen, Taija Finni, Christophe Delecluse, Evelien Van Roie, Timo Rantalainen

**Affiliations:** 1 Faculty of Sport and Health Sciences, Gerontology Research Center, University of Jyväskylä, 40014 Jyväskylä, Finland; laura.i.karavirta@jyu.fi (L.K.); erja.portegijs@jyu.fi (E.P.); kaisa.m.koivunen@jyu.fi (K.K.); taina.rantanen@jyu.fi (T.R.); timo.rantalainen@jyu.fi (T.R.); 2 Physical Activity, Sports and Health Research Group, Department of Movement Sciences, KU Leuven, 3000 Leuven, Belgium; christophe.delecluse@kuleuven.be (C.D.); evelien.vanroie@kuleuven.be (E.V.R.); 3 Faculty of Sport and Health Sciences, Neuromuscular Research Center, University of Jyväskylä, 40014 Jyväskylä, Finland; taija.m.juutinen@jyu.fi

**Keywords:** test–retest, mobility limitation, chair rise

## Abstract

(1) Background: The purpose of this study was to evaluate the day-to-day variability and year-to-year reproducibility of an accelerometer-based algorithm for sit-to-stand (STS) transitions in a free-living environment among community-dwelling older adults. (2) Methods: Free-living thigh-worn accelerometry was recorded for three to seven days in 86 (women *n* = 55) community-dwelling older adults, on two occasions separated by one year, to evaluate the long-term consistency of free-living behavior. (3) Results: Year-to-year intraclass correlation coefficients (ICC) for the number of STS transitions were 0.79 (95% confidence interval, 0.70–0.86, *p* < 0.001), for mean angular velocity—0.81 (95% ci, 0.72–0.87, *p* < 0.001), and maximal angular velocity—0.73 (95% ci, 0.61–0.82, *p* < 0.001), respectively. Day-to-day ICCs were 0.63–0.72 for number of STS transitions (95% ci, 0.49–0.81, *p* < 0.001) and for mean angular velocity—0.75–0.80 (95% ci, 0.64–0.87, *p* < 0.001). Minimum detectable change (MDC) was 20.1 transitions/day for volume, 9.7°/s for mean intensity, and 31.7°/s for maximal intensity. (4) Conclusions: The volume and intensity of STS transitions monitored by a thigh-worn accelerometer and a sit-to-stand transitions algorithm are reproducible from day to day and year to year. The accelerometer can be used to reliably study STS transitions in free-living environments, which could add value to identifying individuals at increased risk for functional disability.

## 1. Introduction

Sit-to-stand (STS) transitions are necessary in daily living [1] and a good STS ability is an important factor in maintaining functional independence [2]. Accordingly, the sit-to-stand test is part of the short physical performance battery (SPPB), widely utilized for capacity assessments among older adults [2]. However, performance technique used and measured in the laboratory may differ from free-living [3,4] and, accordingly, it has been noted that maximal physical performance does not necessarily equate with functioning in daily activities [5]. One of the reasons for this discrepancy is that laboratory measurements cannot take into account the effect of the environment and individual factors on mobility in free-living environments [5]. Therefore, identifying sit-to-stand transitions (STS) in a free-living environment may provide added value to an otherwise laboratory-bound comprehensive performance assessment.

The kinematics of the STS transitions have been measured in many, typically laboratory-bound, studies [6,7,8,9,10,11,12]. For example, a smartphone acceleration sensor has been used to quantify STS transition and was found to be valid [6,7]. The widely used 5x STS test [8,9,10], 10x STS test [11] and sit-to-walk [12] movement kinematics of the different phases have also been interpreted successfully with body-fixed gyroscope and/or accelerometer sensors. In addition, previous studies have measured the power of the STS transitions using force platforms [9] and magnetic-field sensors [13].

Many of the approaches utilized in the laboratory are not feasible in free-living environments due to not being portable or energy requirements being too high for multiple-day recordings. Accelerometers, on the other hand, may provide a feasible alternative for free-living STS assessments. Accelerometers are routinely used to monitor physical activity and functioning in free-living environments over multiple days [14,15]. Body postures and types of physical activity have been reliably identified using wearable triaxial accelerometers [16,17] and STS transitions have been identified in free-living environments using wearable sensors [16,18,19]. Both the number of STS transitions (volume) as well as the intensity of the transitions have been studied previously [20]. However, the reproducibility of STS transition detection and quantification remains to be established.

Reproducibility is the minimum requirement for any assessment to be useful and, therefore, it needs to be determined for identifying and quantifying sit-to-stand transitions (STS) as well. Low reproducibility (low ICC) indicates a random measurement error [21]. The reproducibility of free-living accelerometry-based physical behavior has been estimated for a number of metrics, both in the time scale of day to day and year to year. The reproducibility of accelerometer-assessed physical activity and sedentary behavior has been assessed in older adults [22], children [23,24], and working-age individuals [25]. In addition, reproducibility of accelerometers to detect standing and sitting postural changes [26] and test–retest reliability of the number of STS transitions among type 2 diabetics (64.9 (6.0) years) using the ActivPal [27] and a multiple sensor system [28] among older adults with dementia have been examined. However, the reproducibility of free-living STS transition intensity remains unevaluated among older individuals. This is of practical importance because STS transitions could be monitored in prolonged follow-up studies as an indicator of functional deficits.

The purpose of the present study was to evaluate the day-to-day variability and year-to-year reproducibility of a novel STS volume detection and intensity quantification algorithm in a free-living environment among community-dwelling older adults.

## 2. Materials and Methods

### 2.1. Study Design and Participants

To test the reproducibility of the STS detection and quantification algorithm, data from participants of the AGNES (Active Ageing—Resilience and external support as modifiers of the disablement outcome) counselling intervention study were used (75 and 80 years of age). The study protocol has been published by Rantanen et al. [29,30] and the study was approved by the ethical committee of the Central Finland Health Care District.

The counselling intervention did not affect physical activity and therefore the data from the intervention and control group were pooled for the present study [31]. This resulted in 86 multiple-day (3–7 days) recordings that were repeated with a one-year interval. The baseline records were obtained between October 2017 and August 2018 (baseline), while the one-year follow-up records were obtained between October 2018 and August 2019.

### 2.2. Measurements

Age and sex were extracted from the Digital and Population Data Services Agency register, while height (stadiometer), weight (digital scale Seca, Hamburg, Germany), life-space mobility and cognitive function test (mini-mental state examination, MMSE) were assessed using standardized procedures [30]. Self-reported habitual physical activity was assessed using the Yale Physical Activity Survey for older adults (eight-item, YPAS). The total score range was 0 to 137 and higher scores indicate a higher level of physical activity [32].

Lower-extremity physical performance was assessed in the participants’ homes by the Short Physical Performance Battery (SPPB) [33,34]. The battery comprised tests on standing balance, walking speed over a 3 m distance, and the 5x STS test. In this study, we used the SPPB total score and the time of the 5x STS test as outcomes. Maximal isometric handgrip force was measured on the dominant side during the home interview using a hand-held adjustable dynamometer (Jamar Plus digital hand dynamometer, Patterson Medical, Cedarburg, WI, USA), and expressed in kg [35].

### 2.3. Accelerometry Outcomes

Accelerometry was conducted with a thigh-worn accelerometer (tri-axial accelerometer, which sampled continuously at 100 Hz, 13-bit analog-to-digital conversion, acceleration range ±16 g, UKK RM42, UKK Terveyspalvelut Oy, Tampere, Finland) attached on the anterior aspect of the dominant thigh. 

**Figure 1:**
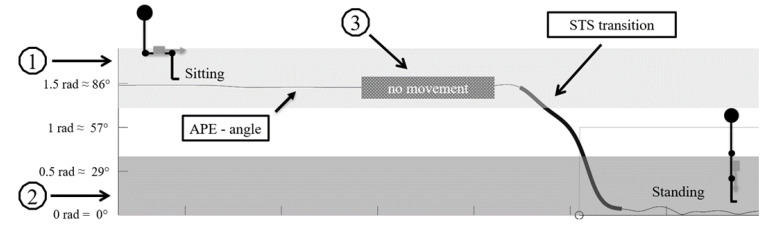
STS transition detection conditions: (1) STS start position > 65 degrees; (2) STS start position > 35 degrees; (3) no motion before STS transition (results of MAD variation < 0.02).

**Figure 2:**
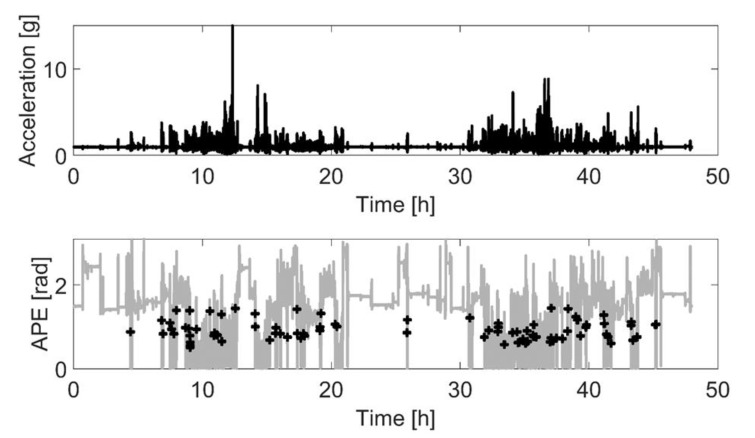
A visualization of the signals used to detect and quantify sit-to-stand (STS) transitions in a free-living environment. The two-day sample starts at midnight and shows the expected diurnal pattern in STS transitions, with very few occurring over the night-time. Top pane: resultant magnitude acceleration. Bottom pane: angle for postural estimation (APE, grey) and the identified STS transitions (black “+”).

The STS transition algorithm (Supplementary Material) was developed using Matlab (R2019a, The MathWorks Inc., Natick, MA, USA). The raw accelerometer data were used to calculate the resultant acceleration for each sampling instant and then the mean amplitude deviation (MAD) was calculated in non-overlapping 5 s epochs [36]. After this, an upright position that serves as a reference vector was determined by searching the data for a walking period that allowed the calculation of the reference posture. Next, the angle for posture estimation (APE) [37] was calculated for each time instant as the vector angle between the reference vector and instantaneous acceleration vector, which had been low-pass filtered at 1 Hz cut-off (4th order zero-lag digital Butterworth filter). The APE signal was further smoothed with a 4th-order Butterworth zero-lag low-pass filter with a 10 Hz cut-off frequency. STS transitions were identified according to the following conditions: (1) movement begins at an APE of at least 65 degrees and ends at an APE of at least 35 degrees; (2) the participant had been stationary for at least 2 s prior to the transition); (3) movement begins at a femoral angle of at least 65 degrees and ends at a femoral angle of at least 35 degrees (Figure 1). The intensity of an identified STS transition was estimated based on the APE signal time derivative (i.e., angular velocity). The STS transition’s mean intensity (mean median angular velocity) was the mean of daily median transitions and the maximal intensity (maximal angular velocity) was defined as the median of the ten fastest STS transitions over the entire monitoring period. The volume of the STS transitions was determined as the number of transitions per monitoring day (Figure 2 and Figure S1).

Physical activity was evaluated from the multiple-day accelerometry records as the daily average mean amplitude deviation (MAD) analyzed in 5 s epochs [38]. In addition, moderate-to-vigorous physical activity (MVPA) minutes were estimated as the daily sum of minutes above 0.24 g MAD. We have previously used the 0.24 g cut-point for high-pass filtered vector magnitude (HPFVM) [39]. MAD and HPFVM calculations resulted in nearly identical numerical values and we therefore deemed it appropriate to apply the HPFVM-based cut-point to MADs for the MVPA analysis.

### 2.4. Statistical Analyses

Results of STS transitions are reported as mean and standard deviation (SD). Shapiro–Wilk normality test was used to check the normality of the data, which indicated that some of the variables were not normally distributed and non-parametric tests were therefore chosen for all variables. The change from baseline to follow-up measurements was analyzed using Wilcoxon signed-rank test and correspondence between the two time points was evaluated with two-way random intraclass correlation coefficients (ICC, absolute agreement, single measures). Agreement between test and retest was analyzed by Bland–Altman analysis [40], where the limits of the agreement were presented with a 95% confidence interval (dotted line). 

In the day-to-day variability analysis, the variation between the five measurement days was examined using the Friedman test (non-parametric Repeated Measures ANOVA). The day-to-day agreement was estimated by calculating intraclass correlation coefficients for four day-pairs (day 1–day 2, day 2–day 3, day 3–day 4 and day 4–day 5) in follow-up measurements. ICC was used to characterize the correspondence as poor (<0.40), fair (0.40 to <0.60), good (0.60 to <0.75) or excellent (≥0.75) [41]. Statistical significance was set at *p* ≤ 0.05 and analyses were performed in the “R” statistical environment (version 4.0.3, R Core Team (2020) [42]. 

The smallest amount of change for STS variables was estimated by calculating the Minimum Detectable Change (MDC) over a 95% confidence interval. This was calculated using the following equations [43]. First, the Standard Error of Measurement (SEM) was calculated:

$${\mathrm{SEM}=\mathrm{SD}}_{1\mathrm{st}\;\mathrm{test}\;}\times \;\sqrt{1-\mathrm{ICC}}$$

The Minimal Detectable Change (MDC) was then calculated for a 95% confidence interval:

$${\mathrm{MDC}}_{95}=1.96\;\times \;\mathrm{SEM}\;\times \;\sqrt{2}$$

## 3. Results

Time taken to complete the 5x STS tests improved from 11.9 (±2.9) seconds at the baseline to 10.3 (±3.0) at the follow-up (*p* = 0.001), with a concomitant improvement in the SPPB total score (10.7 ± 1.4 versus 11.3 ± 1.0, *p* < 0.001) at follow-up (Table 1). No statistically significant changes were observed in hand grip force (*p* = 0.570) or life-space mobility score (*p* = 0.515). In addition, no difference was observed in 24 h mean MAD (*p* = 0.835) nor in MVPA (*p* = 0.567), but self-reported habitual physical activity scores were higher at follow-up (*p* = 0.001).

The mean number of STS transitions at baseline and at follow-up were similar (44.2 ± 15.9 versus 44.5 ± 15.2, *p* = 0.931) (Table 2). Likewise, baseline and follow-up mean angular velocities did not differ (56.9 ± 8.0°/s versus 56.6 ± 8.0°/s, *p* = 0.587). Maximal angular velocity decreased over the follow-up (111.6 ± 22.0°/s versus 107.3 ± 19.7°/s, *p* = 0.017). Physical activity indicated by the 24 h mean MAD (25.1 ± 8.1°/s versus 24.8 ± 8.8°/s, *p* = 0.835) or minutes accumulated in MVPA (34.4 ± 24.7°/s versus 33.7 ± 25.8°/s, *p* = 0.567) did not differ between baseline and follow-up measurements.

The year-to-year ICC’s for the number of STS transitions, mean and maximal angular velocities were good to excellent, i.e., ICC = 0.79 (95% ci 0.70–0.86, *p* < 0.001), ICC = 0.81 (95% ci 0.72–0.87, *p* < 0.001) and ICC = 0.73 (95% ci 0.61–0.82, *p* < 0.001), respectively. The ICCs for MAD (ICC = 0.89, 95% ci 0.84–0.93, *p* < 0.001) and MVPA (ICC = 0.85, 95% ci 0.79–0.90, *p* < 0.001) were excellent. Minimum detectable change (MDC) based on a 95% confidence interval was 20.1 transitions/day for volume, 9.7°/s for mean intensity, and 31.7°/s for maximal intensity.

**Figure 3:**
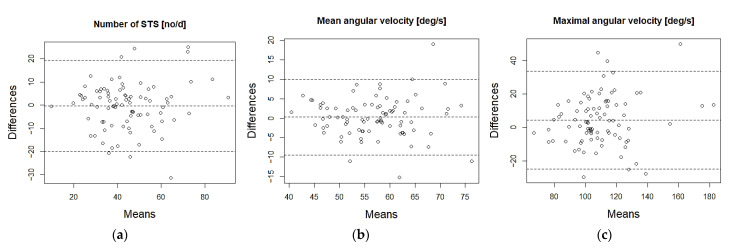
Bland–Altman plots: (**a**) number of STS, (**b**) mean angular velocity and (**c**) maximal angular velocity (*n* = 86).

Day-to-day ICC varied in number of STS transitions between 0.63and 0.72 (95% ci, 0.49–0.81, *p* < 0.001) and in mean angular velocity between 0.75 and 0.80 (95% ci, 0.64–0.87, *p* < 0.001) (Table 3). In addition, there were no statistically significant differences between days in the number of STS transitions (χ^2^(4) = 7.521, *p* = 0.111) and in mean angular velocity (χ^2^(4) = 6.760, *p* = 0.149). Bland–Altman’s analysis (Figure 3) shows that there were only a few cases outside the limits of the agreement (95%, dotted line) and there was no systematic difference between the two measurements.

## 4. Discussion

The purpose of this study was to evaluate the day-to-day variability and year-to-year reproducibility of an accelerometer-based algorithm to detect STS volume and to quantify STS intensity in free-living environments. The results of this study suggest that the algorithm can reliably detect and quantify sit-to-stand transitions in community-dwelling older adults over a year-long follow-up. In addition, the study found a low day-to-day variation in STS volume and intensity. This suggests that STS transitions can be detected, and their intensity quantified reproducibly using a thigh-worn accelerometry in a free-living environment.

The results of the one-year follow-up are congruent with the reproducibility of the number of STS transitions reported for participants with type 2 diabetes, where an excellent agreement (ICC = 0.90, 95% CI 0.79–0.95, *p* < 0.001) has been reported for measurements with at least a one-week time interval [27]. Our study reported better reproducibility of STS transitions than previous reports on the reproducibility of time spent sitting or standing, which showed ICC values of 0.58 for sitting and 0.62 for standing 6 months apart [26]. In addition, the year-to-year ICC values observed in this study are well in line with previously published results when looking at the conventional variables of physical activity. Reproducibility measured with accelerometers has been found to be good between two MVPA measurements among older people within a period of 2–3 years [22], and middle-aged women within a period of 12 months [25]. We are not aware of previous research on the reproducibility of sit-to-stand transition intensity in a free-living environment among older adults, but the test–retest reliability of laboratory-assessed 5x STS test power has been found to be comparable to that of this study [44]. 

In the current study, day-to-day variability was lower in the intensity of STS transitions than in volume. Abel and colleagues (2019) found slightly higher ICC values than this study, when they studied the variation of STS transitions volume from day to day among older people with dementia using a multi-sensor system [28]. We are not aware of any previous study regarding the day-to-day variability of the intensity of STS transitions. A higher variability in the number of STS transitions compared to the intensity may purely suggest that the number of transitions varies in everyday life more than the intensity, which is more dependent on the performance of individuals. To the authors’ best knowledge, minimal detectable change has not been reported previously for STS transitions in free-living environments among older adults. However, in a previous study [43], similar MDC values were observed in the angular velocities of trunk flexion and extension while standing up as in this study. The importance of the minimal detectable change in physical performance assessment should be addressed in future studies.

In this study, participants’ lower extremity performance (SPPB), 5x STS test results and self-reported physical activity (YPAS) improved during one year of follow-up. On the other hand, life-space mobility, physical activity monitored by an accelerometer and hand grip force did not change during one-year follow-up. Reviewing other questionnaire data recorded in the trial not reported in this study [29,30] indicated that none of the individuals that took part in the follow-up had a major health-related or psychological setback during the follow-up. In addition, physical activity measured with an accelerometer was concordant with STS variables, i.e., no change over the follow-up, which could indicate that physical behavior had not changed significantly. Therefore, the very good-to-excellent year-on-year agreement of the STS transition assessment seems encouraging and thus the assessment provides a reliable method to measure the volume and intensity of STS transitions in free-living environments. 

The presented detection algorithm contains a few limitations that need to be pointed out. The algorithm is only able to identify the first repetition of a multi-STS set (caused by the 2 s stationary epoch prior to an STS requirement) and therefore cannot be directly used to identify, e.g., the performance in the 5x STS. The algorithm can also detect movements other than STS transitions, such as very slow knee lifts or other similar movements where the hip joint is flexed and extended toward the ground at a slow pace. However, the algorithm contains a criterion that requires that there must be no movement before the STS transition, and therefore there should be no interference with other activity types, such as walking, running or climbing stairs. Nevertheless, STS transition detection sensitivity and specificity would need to be examined more rigorously in future studies. The presented algorithm does not require aligning the sensor in any specific orientation with respect to the thigh because the orientation is estimated from the signal. In this study, the equipment was not calibrated separately because the calibration performed by the equipment manufacturer was of sufficient quality. If applying the algorithm to data recorded using a device with marked calibration imprecision, e.g., the autocalibration method proposed by Van Hees and colleagues [45], there is sufficient calibration according to our experimentation.

This study has some limitations that need to be pointed out. Although the year-to-year stability is encouraging, significant changes in the physical performance of an older person may take place over a year-long follow-up [46,47]. Therefore, it was impossible to disentangle physiological changes over the follow-up from the imprecision associated with the measurement. However, no change in physical activity was observed between the two measurements. In addition, the validity of the STS intensity evaluation algorithm remains to be investigated. The strength of this study is that it included a relatively large sample of community-dwelling participants with multiple days (3–7 days) of recording accelerometry. The 3–7-day accelerometry sample is thought to be sufficient for assessing activity patterns [48]. The developed algorithm is independent of the measurement device and can be applied to raw accelerations recorded with any reasonably precise accelerometer. The year-to-year reliability of the STS transition detection and quantification appeared to be acceptable, and we postulate it would be reasonable to apply the technique in further studies. For example, the convergent validity of free-living STS detection and quantification could be evaluated by exploring the associations between laboratory-measured performance capacity and free-living STS transition quantifications.

## 5. Conclusions

This study provided evidence supporting long-term and day-to-day reproducibility of accelerometer-measured STS volume detection and an intensity quantification algorithm in free-living environment community-dwelling older adults. The algorithm can be used to reliably study STS transitions in free-living environments, which could add value to identifying individuals at increased risk for functional disability.

## Figures and Tables

**Table 1:** Descriptive statistics of the study (*n* = 86, female 64%) (mean (SD)).

	Baseline	Follow-Up	*p*-Value ^1^
Age [year]	76.5 (±1.9)		
Weight [kg]	73.7 (±14.0)		
Height [m]	164.6 (±9.8)		
MMSE [points]	28.2 (±1.3)		
YPAS [points]	57.7 (±21.0)	66.6 (±24.6)	0.001
Life-space mobility [points]	74.2 (±10.3)	75.3 (±14.1)	0.515
Hand grip force [kg]	35.3 (±11.3)	36.7 (±12.7)	0.570
5x STS test time [s]	11.9 (±2.9)	10.3 (±3.0)	<0.001
SPPB overall points [points]	10.7 (±1.4)	11.3 (±1.0)	<0.001
MAD 24 h [mG]	25.1 (±8.1)	24.8 (±8.8)	0.835
MVPA [min/d]	34.4 (±24.7)	33.7 (±25.8)	0.567

SD = standard deviation; MMSE = Mini-Mental State Examination; YPAS = self-reported habitual physical activity scores from the Yale Physical Activity Survey for older adults; STS = sit-to-stand; SPPB = Short Physical Performance Battery; MAD = mean amplitude deviation; MVPA = moderate-to-vigorous physical activity; ^1^ Wilcoxon signed-rank test.

**Table 2:** Sit-to-stand assessment from the free-living recordings at baseline and the one-year follow-up and reproducibility of the sit-to-stand transition outcomes (*n* = 86).

	Baseline Mean (SD)	Follow-Up Mean (SD)	*p*-Value ^1^	ICC	ICC 95% ci
Number of STS [no/d]	44.2 (±15.9)	44.5 (±15.2)	0.931	0.79 ***	0.70–0.86
Mean angular velocity [deg/s]	56.9 (±8.0)	56.6 (±8.0)	0.587	0.81 ***	0.72–0.87
Maximal angular velocity [deg/s]	111.6 (±22.0)	107.3 (±19.7)	0.017	0.73 ***	0.61–0.82
MAD 24 h [mg]	25.1 (±8.1)	24.8 (±8.8)	0.835	0.89 ***	0.84–0.93
MVPA [min/d]	34.4 (±24.7)	33.7 (±25.8)	0.567	0.85 ***	0.79–0.90

STS = sit-to-stand; SD = standard deviation; ICC = intraclass correlation coefficients; Ci = confidence interval of ICC; ^1^ Wilcoxon signed-rank test; *** *p* < 0.001.

**Table 3:** Mean values of free-living STS variables for five follow-up recording days and intraclass correlation coefficients between four day-pairs (*n* = 86).

Mean (SD)	Number of STS (no/day)	Mean Angular Velocity (deg/s)
day 1 (*n* = 85)	45.5 (±17.7)	56.2 (±8.1)
day 2 (*n* = 86)	44.4 (±16.1)	55.5 (±8.3)
day 3 (*n* = 86)	44.4 (±20.0)	57.2 (±7.9)
day 4 (*n* = 83)	43.4 (±17.4)	56.4 (±9.2)
day 5 (*n* = 81)	45.9 (±18.2)	56.8 (±8.6)
ICC (95% ci) ***		
day 1–day 2 (*n* = 85)	0.63 [0.49, 0.74]	0.79 [0.69, 0.86]
day 2–day 3 (*n* = 86)	0.72 [0.60, 0.81]	0.78 [0.68, 0.86]
day 3–day 4 (*n* = 83)	0.64 [0.50, 0.75]	0.75 [0.64, 0.83]
day 4–day 5 (*n* = 81)	0.71 [0.58, 0.80]	0.80 [0.71, 0.87]

STS = sit-to-stand; SD = standard deviation; ICC = intraclass correlation coefficients; ci = confidence interval of ICC; *** all *p* < 0.001.

## Data Availability

After completion of the study, data will be stored at the Finnish Social Science Data Archive without potential identifiers (open access). Until then, pseudonymized datasets are available to external collaborators subject to agreement on the terms of data use and publication of results. To request the data, please contact Professor Taina Rantanen (taina.rantanen@jyu.fi).

## Supplementary Materials

The following are available online at https://www.mdpi.com/article/10.3390/s21186068/s1, Figure S1: A visualization of the signals used to detect and quantify sit-to-stand (STS) transitions in a free-living environment. Video S1: Sit-to-stand detection and quantification algorithm sample is available at https://cmj.sport.jyu.fi/sittostand/. Accelerometer-based sit-to-stand transitions algorithm is available at https://github.com/tjrantal/SitToStandSupplement (accessed on 13 June 2021).
