# Supplementary material for: Day-to-Day Variability and Year-to-Year Reproducibility of Accelerometer-Measured Free-Living Sit-to-Stand Transitions Volume and Intensity among Community-Dwelling Older Adults
Source: Sensors (Basel). 2021 Sep 10;21(18):6068. doi: 10.3390/s21186068 (PMC8471908; doi:10.3390/s21186068)
Supplement: Supplementary file 1 [file sensors-21-06068-s001.zip › sensors-1279025-supplementary.pdf]

Supplementary material 1
Löppönen et al. 2021

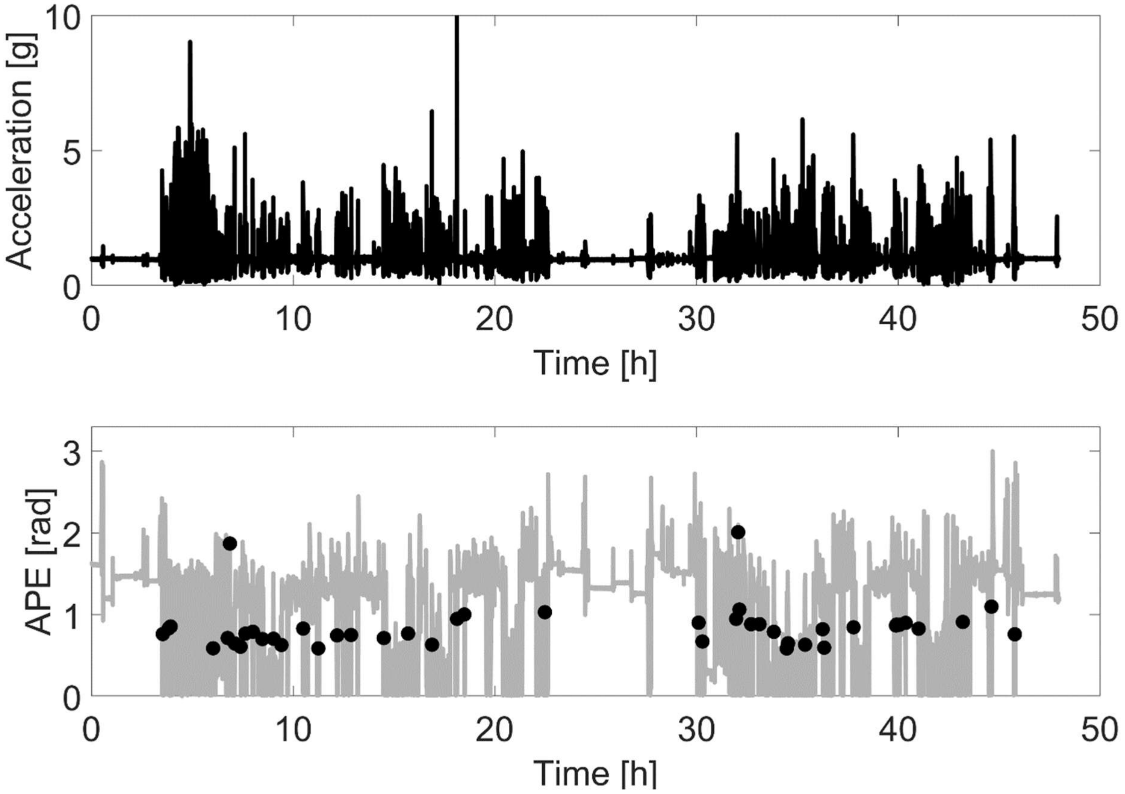

Large number of STS transitions – baseline

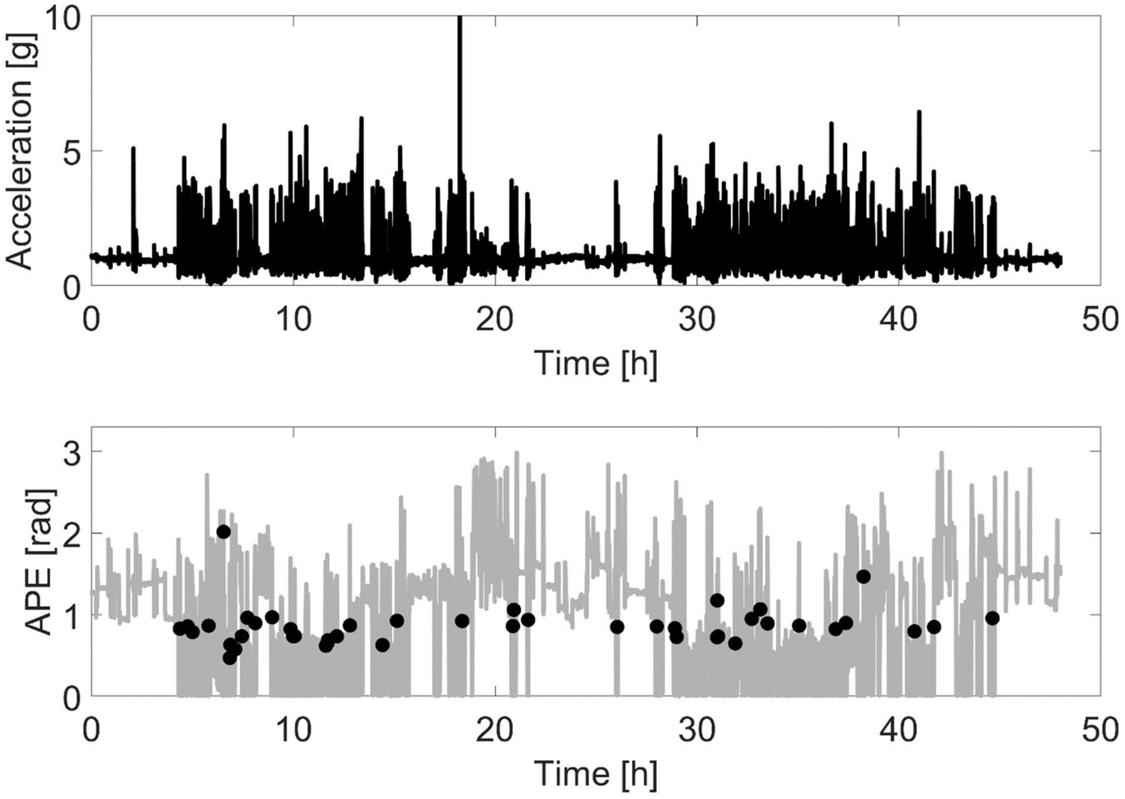

Large number of STS transitions – follow-up

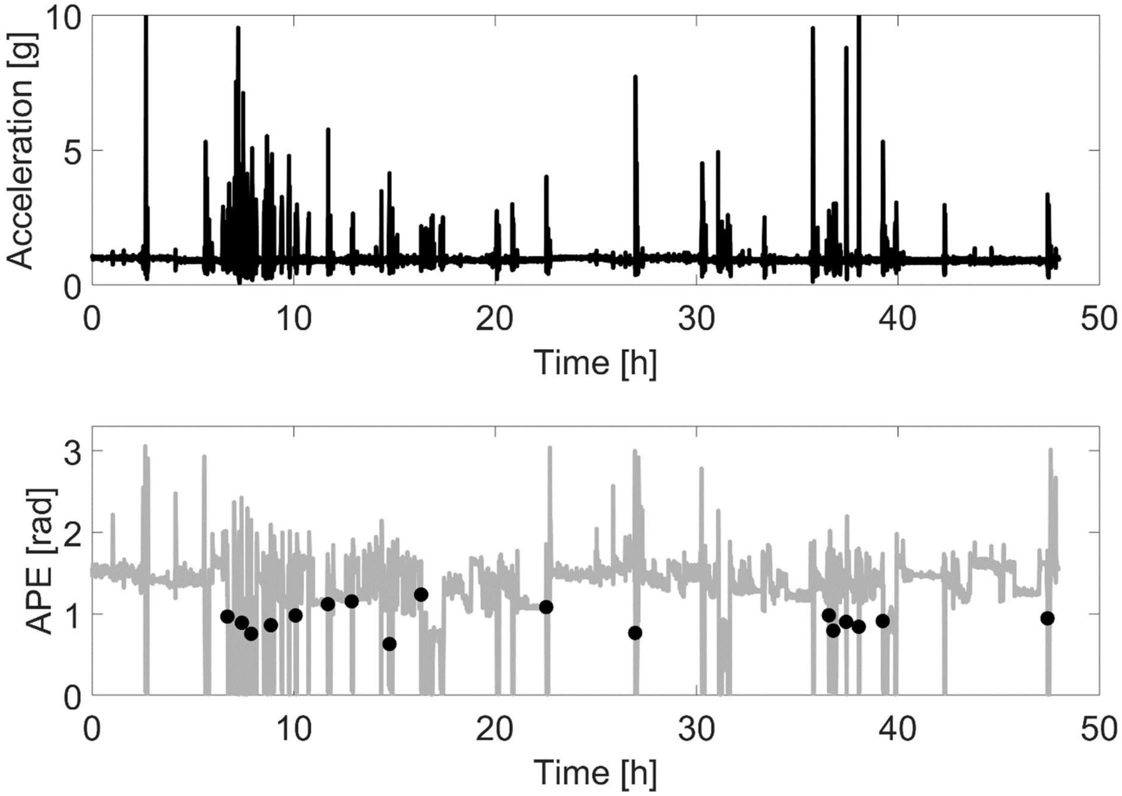

Small number of STS transitions – baseline

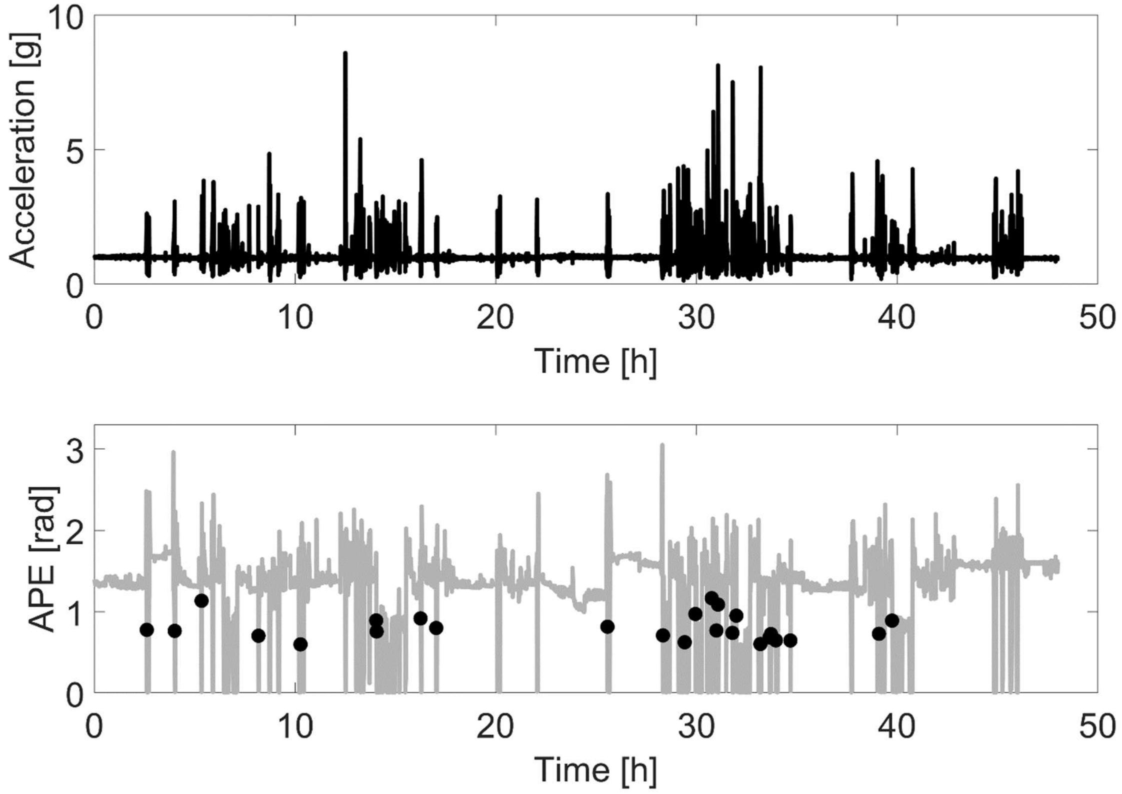

Small number of STS transitions – follow-up
